# Application of a Bayesian graded response model to characterize areas of disagreement between clinician and patient grading of symptomatic adverse events

**DOI:** 10.1186/s41687-018-0086-x

**Published:** 2018-12-04

**Authors:** Thomas M. Atkinson, Bryce B. Reeve, Amylou C. Dueck, Antonia V. Bennett, Tito R. Mendoza, Lauren J. Rogak, Ethan Basch, Yuelin Li

**Affiliations:** 1 Department of Psychiatry & Behavioral Sciences, Memorial Sloan Kettering Cancer Center, 641 Lexington Ave., 7th Floor, New York, NY 10022 USA; 2 Duke University Medical Center, Durham, NC USA; 3 Mayo Clinic, Scottsdale, AZ USA; 4 University of North Carolina at Chapel Hill, Chapel Hill, NC USA; 5 University of Texas M.D. Anderson Cancer Center, Houston, TX USA

**Keywords:** Patient-reported outcomes, Clinician-patient agreement, Item response theory, Neoplasms

## Abstract

**Background:**

Traditional concordance metrics have shortcomings based on dataset characteristics (e.g., multiple attributes rated, missing data); therefore it is necessary to explore supplemental approaches to quantifying agreement between independent assessments. The purpose of this methodological paper is to apply an Item Response Theory (IRT) -based framework to an existing dataset that included unidimensional clinician and multiple attribute patient ratings of symptomatic adverse events (AEs), and explore the utility of this method in patient-reported outcome (PRO) and health-related quality of life (HRQOL) research.

**Methods:**

Data were derived from a National Cancer Institute-sponsored study examining the validity of a measurement system (PRO-CTCAE) for patient self-reporting of AEs in cancer patients receiving treatment (*N* = 940). AEs included 13 multiple attribute patient-reported symptoms that had corresponding unidimensional clinician AE grades. A Bayesian IRT Model was fitted to calculate the latent grading thresholds between raters. The posterior mean values of the model-fitted item responses were calculated to represent model-based AE grades obtained from patients and clinicians.

**Results:**

Model-based AE grades showed a general pattern of clinician underestimation relative to patient-graded AEs. However, the magnitude of clinician underestimation was associated with AE severity, such that clinicians’ underestimation was more pronounced for moderate/very severe model-estimated AEs, and less so with mild AEs.

**Conclusions:**

The Bayesian IRT approach reconciles multiple symptom attributes and elaborates on the patterns of clinician-patient non-concordance beyond that provided by traditional metrics. This IRT-based technique may be used as a supplemental tool to detect and characterize nuanced differences in patient-, clinician-, and proxy-based ratings of HRQOL and patient-centered outcomes.

**Trial registration:**

ClinicalTrials.gov NCT01031641. Registered 1 December 2009.

## Background

Levels of concordance, specifically the degree to which two or more individuals agree when independently rating something such as the severity of pain, can be calculated using a number of different statistical metrics (e.g., Cohen’s weighted κ, Spearman’s *r*) [1, 2]. While each of these statistical tests allow for a single coefficient to quantify concordance, they do not reflect the degree to which these levels of agreement may differ based on the variability of the individual(s) assigning the rating (e.g., a clinician or patient). Additionally, the characteristics of the dataset of interest, including the type of response scale used, the base-rate of responses (i.e., proportion of positive, negative or zero values in the sample), and the ratio of raters to items can impact the degree to which these traditional metrics can be confidently interpreted as being accurate [3, 4]. In our research comparing patients’ self-reports with providers’ ratings of symptom severity using an ordinal response scale, we have seen the inter-rater agreement highly dependent on the prevalence of the symptom; a high proportion of 0–0 (i.e., None – None) pairs of ratings will cause the statistics to show an inflated level of agreement when that may not be the case among the subset of patients who experienced the symptom [5].

**Fig. 1:**
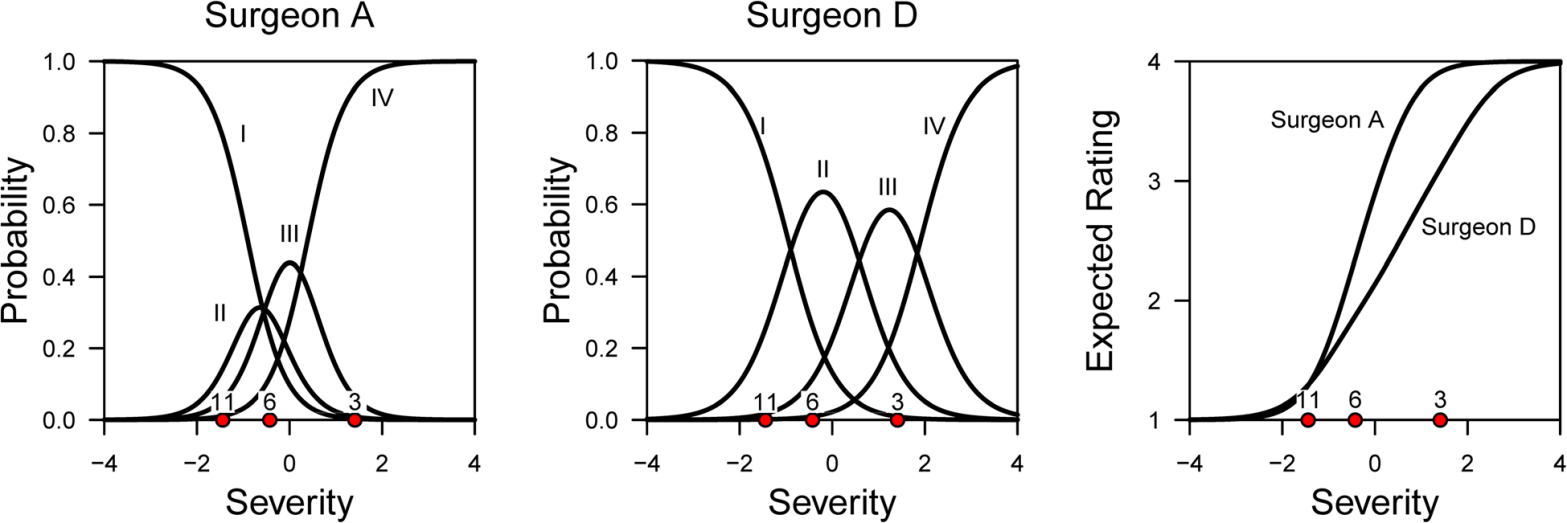
Graded Response Model tracelines depicting two orthopedic surgeons’ responses in classifying hip fracture severity. *Note:* Posterior mean severity locations of three patients are superimposed. Plot recreated using published data [6]

A supplemental approach to the calculation of concordance was proposed by Baldwin [6] using a Graded Response Model (GRM) to explicitly model the item response probability. In this example, independent orthopedic surgeons made use of a four-level severity classification to review radiographs and rate patient hip fracture severity. A Bayesian GRM was applied, in which surgeons were treated like items in GRM so that the item parameters were taken to represent differences between surgeons’ internal decision criteria. Figure 1 illustrates the subtle differences between two orthopedic surgeons. The leftmost plot shows the four tracelines of the probability of surgeon A’s classifications for the four hip fracture severity levels. As the underlying severity of a hip fracture increases, the likelihood of a patient being graded with a higher classification (from Type I to Type IV fracture) increases. The model-predicted severity for patients’ radiographs 11, 6, and 3 are superimposed, (i.e., for surgeon A, radiograph 11 is expected to be rated minor (Type I), radiograph 6 is likely to be judged Type III, and radiograph 3 as Type IV). For surgeon D, however, the three radiographs are expected to be rated Type I (which agrees with surgeon A), category II and III, respectively. The tracelines are analogous to the conventional item characteristic curves in IRT models, and the item difficulty (i.e., threshold) parameters in the fitted GRM model represented the surgeons’ decision cutoffs and the item discrimination represented how sensitive the surgeons’ responses were with respect to changes in hip fracture severity. Their model-based, GRM approach differs from conventional methods in that it can identify subtle but important differences between raters (e.g., discordance only emerges at higher levels of latent hip fracture severity).

Inter-rater disagreement arises because raters differ in their underlying decision thresholds in a GRM model. Such a model-based approach offers several advantages. Raters may differ generally (e.g., some surgeons tend to assign more severe ratings to most cases). Rater discordance may also be contextual (e.g., surgeons may differ in how they rate specific cases). These subtle differences are important in eliciting patient-reported outcomes (PROs) in a clinical encounter, where, for example, a patient may report a mild pain to a clinician but later endorse ‘moderate’ pain when given an assessment survey. Our approach is in principle similar to other IRT-based approaches to inter-rater differences (e.g., many-facet Rasch model) [7].

We have previously successfully applied this Bayesian GRM framework in our own research to explore differences between patient-, clinician-, and nurse-based ratings of symptomatic adverse events (AEs) in the clinical trial setting [8]. However, the data used in that study included a limited number of unidimensional ratings of AEs, and were from a single, tertiary cancer center with limited patient diversity with respect to demographics and disease type.

The present study builds upon our application of the Bayesian GRM and explores the concordance between unidimensional clinician ratings of patients’ AEs (e.g., nausea, diarrhea, fatigue), and the patients’ self-report of multiple attributes (e.g., frequency, severity, interference with daily activities) of those same symptomatic AEs during cancer treatment. In this context, concordance refers to the agreement between expected ratings, given the same underlying level of an underlying symptoms. We also sought to demonstrate that IRT has potential applicability in probing concordance in similar datasets that involve two or more independent raters of an event, particularly in patient-centered outcomes research where raters may include patients, caregiver proxies, and clinicians.

### Dataset context

In the United States, AEs are monitored as part of all cancer clinical trials for the purposes of understanding treatment-related toxicities and ensuring patient safety. While currently these AEs are documented by clinicians, there are emerging efforts to directly capture the patient experience of symptomatic AEs using PROs for those AEs that represent symptoms [9]. This has led to a number of studies that have directly compared the concordance between clinician and patient symptomatic AE ratings [10, 11], with the majority of these studies demonstrating that AE ratings tend to be discordant [12, 13]. However, the common metrics used for quantifying concordance in AE grading are prone to the aforementioned shortcomings that preclude the isolation of potential sources contributing to this discordance [14–16]. Additionally, in a naturalistic clinic setting, a given clinician is responsible for the care of any number of patients. The variability of the ratings adjudicated by a specific clinician may be more homogeneous than that from independent patients receiving care from the same clinician. This creates a unique analytic challenge when applying the Bayesian GRM in estimating multiple clinicians’ decision thresholds as item difficulties in an IRT model, as there would be missing clinicians’ ratings for patients not seen by a given clinician. These issues highlight the need for a supplemental methodological approach to understanding clinician-patient concordance in AE reporting.

## Methods

### Participants

The study sample for this secondary analysis included 940 patients receiving active treatment for various malignancies and participating in an NCI-sponsored multi-institutional study examining the validity of PRO-CTCAE (Clinical Trials.gov NCT01031641) [17]. English speaking patients were eligible to participate if they were ≥ 18 years of age, were undergoing chemotherapy or radiation therapy for cancer, and were not cognitively impaired. Data were collected between February 2011 and May 2012. The study was approved by the institutional review boards at the National Cancer Institute and all participating sites. All study participants provided written informed consent.

### Measures

*Common Terminology Criteria for Adverse Events version 4(CTCAE* [18]*)* – CTCAE consists of a library of over 700 descriptive terms for clinician-based assessment of patient AEs related to cancer treatment. Each CTCAE term is assessed using a 5-point verbal descriptor grading scale, with each grade following a similar grading convention (i.e., 0 = not present, 1 = mild, 2 = moderate, 3 = severe and/or requiring medical intervention but not life-threatening, 4 = life-threatening consequences, and 5 = death).

*Patient-Reported Outcomes version of the Common Terminology Criteria for Adverse Events (PRO-CTCAE* [17, 19–23]*) –* PRO-CTCAE is an item library comprised of 124 items representing various attributes of 78 discrete CTCAE-derived symptom terms.^1^ Each item uses a 0–4 rating scale that is attribute dependent (i.e., *frequency*: (0) never, (1) rarely, (2) occasionally, (3) frequently, (4) almost constantly*; severity*: (0) none, (1) mild, (2) moderate, (3) severe, (4) very severe; and *interference with daily activities*: (0) not at all, (1) a little bit, (2) somewhat, (3) quite a bit, (4) very much). In the present dataset, 13 PRO-CTCAE symptoms included at least two symptom attributes and a corresponding clinician CTCAE grade: anxiety, dyspnea, edema, fatigue, feelings that nothing could cheer you up, headache, insomnia, mucositis, nausea, pain, problems with concentration, sad or unhappy feelings, vomiting. These 13 symptoms were selected to demonstrate the utility of the Bayesian GRM to characterize the concordance between a clinician’s single, unidimensional rating of a symptomatic toxicity using CTCAE, and the corresponding patient ratings of multiple attributes for that symptom.

### Procedure

Using hand-held computers, patients completed PRO-CTCAE items evaluating the frequency and severity (nausea and vomiting), severity and interference (dyspnea, fatigue, insomnia, mucositis, and problems with concentration), and frequency, severity and interference (anxiety, edema, headache, pain, feelings that nothing could cheer you up, and sad or unhappy feelings) of 13 AEs. The data structure contained conditional branching, such that in instances where respondents assigned a rating of never (0) for the frequency attribute or none for the severity attribute (presented first in the series), the additional attributes were not presented to them and thus skipped, and for the purposes of this analysis, were coded as missing [24]. During that same clinic visit, the same 13 AEs were rated by clinicians using the CTCAE, and documented in the electronic health record. Although PRO-CTCAE and CTCAE rating were obtained concurrently, clinicians did not have access to the PRO-CTCAE responses when assigning their CTCAE scores.

### Statistical analysis

The first step in the Bayesian GRM involved restructuring the raw data. Unlike Baldwin’s study [6], our raw data contained limitations: 1) each patient was rated by one clinician who provided care; and 2) details on the identity of the clinicians were unavailable (i.e., which patient or group of patients were seen by a single clinician at a given study site). To best approximate such clustering in the data structure, a composite variable was created, consisting of all observed unique combinations of institutions and cancer types, which yielded each patient being nested within 45 unique institution/cancer combinations. Thus, the present application of GRM accounted for the decision thresholds associated with these 45 unique clinician clusters, representing clinic-based aggregate reports, hereinafter referred to as “clinics.” Note that within such clinics, a given clinician may have rated one or multiple patients, but no patient was rated by more than one clinician. For example, clinic 2 might refer to “Site 1, Breast Cancer”, whereas clinic 15 might refer to “Site 5, Gastrointestinal Cancer”. Table 1 represents a single symptom example of the data structure in our analysis, with the columns representing scale items fitted. For each column, GRM item discrimination and thresholds were calculated. The posterior mean values of the model-fitted item responses were calculated to represent model-based AE grades obtained from patients and clinics independently.

**Table 1:** Example of data entry structure

	PRO-CTCAE			CTCAE						
Patient ID	Frequency	Severity	Interference	Clinic1	Clinic2	Clinic3	Clinic4	Clinic43	Clinic44	Clinic45
001	1	1	1	0	N/A	N/A	N/A	N/A	N/A	N/A
002	0	–	–	N/A	1	N/A	N/A	N/A	N/A	N/A
003	2	1	2	N/A	N/A	1	N/A	N/A	N/A	N/A
004	1	2	0	N/A	N/A	1	N/A	N/A	N/A	N/A
005	0	–	–	N/A	N/A	0	N/A	N/A	N/A	N/A
006	1	2	3	N/A	N/A	N/A	2	N/A	N/A	N/A
007	2	1	0	N/A	N/A	N/A	0	N/A	N/A	N/A
008	1	1	3	N/A	N/A	N/A	1	N/A	N/A	N/A
935	2	1	1	N/A	N/A	N/A	N/A	1	N/A	N/A
936	0	–	–	N/A	N/A	N/A	N/A	0	N/A	N/A
937	2	2	2	N/A	N/A	N/A	N/A	2	N/A	N/A
938	1	1	2	N/A	N/A	N/A	N/A	1	N/A	N/A
939	2	1	0	N/A	N/A	N/A	N/A	N/A	2	N/A
940	2	2	3	N/A	N/A	N/A	N/A	N/A	N/A	1

*Note: CTCAE* indicates Common Terminology Criteria for Adverse Events, *PRO-CTCAE* indicates Patient-Reported Outcomes version of the Common Terminology for Adverse Events. Clinic# indicates which of the 45 clinics (grouping clinicians within the same clinic) provided the CTCAE grades. N/A indicates data was not collected for a given patient in a given clinic. -- indicates PRO-CTCAE data was not captured for the severity and interference attributes for a given adverse event due to the frequency attribute being assigned a zero (never) rating

As part of any IRT analysis, items are assumed to be locally independent [25]. In this case local independence among patient and clinic ratings was assumed to simplify the illustrative examples provided below, with an additional assumption that there is one single underlying latent variable representing the multiple attributes for a given symptom (i.e., frequency, severity, interference with daily activities). The rating thresholds themselves are not viewed as latent variables – their existence is inferred because of the observed rating values.

Since all clinics did not assess AEs in all patients, instances where a given clinic did not make a rating were treated as missing (noted by “N/A”). For example, individual clinicians in Clinic 4 may have rated patients 006–012 but no other patients in the dataset. The Bayesian GRM approach updates the parameter estimates based on available data only, therefore missing data provides no information with respect to the posterior distributions of the parameters. This permitted the modeling of decision thresholds across the aggregated clinic clusters in an actual clinical encounter, without the need to compel a rectangular data structure. In this analysis we focus on the model-based expected item responses between patients and clinics. This model-based approach is advantageous in that it permits the extraction of core information out of data that contains multiple sources of variability. Model-based responses represent the most likely symptom ratings from patients and clinics with random error variabilities parsed out. The Bayesian GRM assumes the existence of underlying response thresholds [26], with threshold cutoffs treated the same for clinics and patients (e.g., a response of 1 through 4 versus 0, a response of 2 through 4 versus 0 through 1). These parameter estimates provide the internal standards that are used by the raters.

The Bayesian GRM item threshold parameters were given a normal, weakly informative prior with a mean of 1.0 (average slope of 1 in item discrimination) and a standard deviation of 2.5 [27]. The kappa parameters were given a normal prior of 0.0 and a standard deviation of 2.5. All analyses were completed using R version 3.4 [28] and JAGS version 4.3.0 [29],^2^ using shared syntax [30], with 1000 adaptation iterations, 5000 burn-in iterations, and an additional 8000 iterations (after thinning by 10) kept for the posterior parameter estimates.

## Results

Participant characteristics are provided in Table 2. Participants ranged in age 19–91 years (mean = 58 years; standard deviation ±12) with varied cancer types. The sample was predominantly Caucasian (71.8%); 21.6% were Black or African American; 6% were of Hispanic ethnicity. PRO-CTCAE responses had a theoretical range of 0–5, whereas CTCAE ratings had a theoretical range of 0–3.

**Table 2:** Patient characteristics (*N* = 940)

Characteristic	No. of Patients (*N* = 940)		%
Age range		19–91	
Mean, years (±SD)		58.26	
Female	539		57.3
ECOG Performance Status			
Median		1.00	
Disease			
Breast	260		27.7
Lung/Head/Neck	329		35.0
Gastrointestinal	95		10.1
Genitourinary/Gynecologic	172		18.3
Hematological	47		5.0
Other/Unknown	37		3.9
Race			
White	675		71.8
Black or African American	203		21.6
Native Hawaiian/Pacific Islander	5		0.5
Asian	42		4.5
Native American/Pacific Islander	2		0.2
Multiple Reported	13		1.4

*Note: ECOG* indicates Eastern Cooperative Oncology Group

Tables 3 and 4 display an example of the degree to which independent CTCAE and PRO-CTCAE ratings for the symptom of pain vary. Table 3 contains a sample from the larger dataset to illustrate the observed distribution of CTCAE pain grades for those patients who endorsed values of all 1, 2, or 3 for frequency, severity, and interference with daily activities (*n* = 83). The first row of the table illustrates that for the 24 patients in the sample who reported ‘rarely’-occurring, ‘mild’ pain that interfered ‘a little bit’ with daily activities – a majority of the corresponding ratings of pain by clinicians were CTCAE grade 1. The second row shows that ‘occasional’, ‘moderate pain’, that ‘somewhat’ interferes with daily activities was also graded by a majority of the clinicians as CTCAE grade 1. Thus, intuitively, there appears to be good concordance between patients’ and clinicians’ ratings in the setting of pain that occurs ‘rarely’, was ‘mild’, and interfered ‘a little bit’ with daily activities (21 of 24 clinicians rated this CTCAE Grade 1). However there was less agreement among clinicians’ ratings of those patients who reported ‘occasional’, ‘moderate’ pain, that ‘somewhat’ interferes with daily activities, as seen in the 27 ratings of this experience as CTCAE grade 1, and eight clinicians who rated this as CTCAE grade 2 pain. A similar discrepancy was observed with ‘frequent’, ‘severe’ pain, that interferes ‘quite a bit’ with daily activities, where five clinicians assigned a CTCAE grade of 3 and 19 assigned a lower grade (9 grade 1; 10 grade 2).

**Table 3:** Distribution of Raw CTCAE Ratings when PRO-CTCAE Pain ratings all 1, 2, or 3 (*n* = 83)

			CTCAE Pain Grade		
PRO-CTCAE Pain Rating					
Frequency	Severity	Interference	1	2	3
‘rarely’	‘mild’	‘a little bit’	21	3	0
‘occasionally’	‘moderate’	‘somewhat’	27	8	0
‘frequent’	‘severe’	‘quite a bit’	9	10	5

*Note:* This example represents respondents for which CTCAE and PRO-CTCAE pain was captured, and where frequency, severity and interference were rated as all 1, 2, or 3 by patients

**Table 4:** Example of cross-tabulation of CTCAE pain grades and PRO-CTCAE pain severity ratings (*n* = 525)

		CTCAE Pain grade				
PRO-CTCAE Pain severity		0	1	2	3	Total
‘none’	0	2	2	1	0	5
‘mild’	1	112	85	13	0	210
‘moderate’	2	56	92	32	3	183
‘severe’	3	16	33	29	13	91
‘very severe’	4	4	5	21	6	36
	Total	190	217	96	22	525

*Note:* This sample size represents respondents in whom both CTCAE and PRO-CTCAE pain ratings were captured

Table 4 displays the cross-tabulation of clinicians’ CTCAE pain ratings and patients’ PRO-CTCAE pain severity reports. Pain was reported by 525 patients, however, only 132 pairs of CTCAE and PRO-CTCAE ratings fall on the main diagonal entries, suggesting discordance in how patients and clinicians assign their ratings.

Table 5 displays the means, standard deviations of symptom severity; and the traditional concordance measures between CTCAE and PRO-CTCAE. PRO-CTCAE severity was used here for the calculation of concordance since it was the only attribute captured for every AE in the present dataset. The ranges of Cohen’s weighted κ and Spearman’s *r* coefficients (i.e., 0.05–0.41 and 0.13–0.58, respectively) indicate a low to moderate association between CTCAE and PRO-CTCAE severity ratings, while the raw percentages represent unadjusted levels of agreement between these two types of ratings.

**Table 5:** Means, standard deviations, and traditional concordance metrics for patient adverse events

	Mean (Standard Deviation)				Concordance Metric		
	PRO-CTCAE			CTCAE			
Adverse Event	Frequency	Severity	Interference	Grade	Weighted κ	Spearman *r*	%
Anxiety	1.18 (1.08)	1.55 (0.79)	1.07 (1.13)	0.48 (0.66)	0.05	0.36*	0.28
Dyspnea	**	0.69 (0.93)	1.32 (1.13)	0.34 (0.60)	0.41*	0.58*	0.64
Edema	0.57 (1.10)	1.68 (0.88)	1.17 (1.28)	0.16 (0.41)	0.07	0.13	0.22
Fatigue	**	1.68 (1.07)	1.80 (1.14)	0.98 (0.77)	0.25*	0.48*	0.37
Feeling Nothing/Cheer Up	0.67 (0.96)	1.43 (0.83)	1.17 (1.05)	0.30 (0.55)	0.09	0.30*	0.25
Headache	0.74 (0.95)	1.46 (0.71)	0.96 (1.00)	0.20 (0.46)	0.05	0.21*	0.19
Insomnia	**	1.13 (1.12)	1.43 (1.12)	0.51 (0.71)	0.29*	0.50*	0.48
Mucositis	**	0.42 (0.84)	1.09 (1.19)	0.16 (0.49)	0.35*	0.47*	0.77
Nausea	0.86 (1.07)	1.55 (0.86)	**	0.33 (0.56)	0.09	0.31*	0.28
Pain	1.35 (1.29)	1.89 (0.95)	1.62 (1.25)	0.61 (0.79)	0.15	0.44*	0.25
Problems w/Concentration	**	0.71 (0.90)	1.24 (0.98)	0.34 (0.54)	0.30*	0.43*	0.58
Sad/Unhappy Feelings	1.14 (0.99)	1.42 (0.76)	0.94 (1.04)	0.30 (0.55)	0.08	0.39*	0.21
Vomiting	0.29 (0.71)	1.51 (0.94)	**	0.10 (0.36)	0.10	0.34*	0.25

*Note:* Concordance was calculated between PRO-CTCAE severity and CTCAE ratings, ** indicates attribute not assessed, * indicates significant *p* < 0.01

### Graded response model

Overall, the α and κ parameters in a GRM can be conceptualized as the slopes and intercepts, where the slopes represent how item responses are sensitive to the underlying latent construct and the intercepts represent thresholds of consecutive response categories. They offer a crude but practical representation of patients’ and clinics’ underlying decision criteria when they assess pain. Table 6 is a representation of a single AE (i.e., pain), and shows a pattern where patients’ α parameter estimates are generally greater than clinicians’ (as the latent pain increases, patients tend to report greater pain at a faster pace than clinics). As for κ thresholds, patients tend to have lower κ thresholds than clinics in reporting grade 1 and 2 pain. However, when it comes to pain levels greater than 2, patient’s thresholds appear to be comparable to clinics’. The pattern arises from the observations that, by and large, patients tend to report grade 1–2 pain while their clinics report grades 0–1 pain. Pain reports above grade 3, however, are uncommon in both patients’ and clinicians’ reports that they result in roughly comparable GRM estimates.

**Table 6:** Bayesian Graded Response Model Estimates for Pain

	α parameter			κ parameter				
	Frequency	Severity	Interference		1–4 vs. 0	2–4 vs. 0–1	3–4 vs. 0–2	4 vs. 0–3
Patient	5.04	5.69	4.33	Frequency	−2.58	0.41	3.99	6.98
				Severity	−6.46	1.21	5.48	9.10
				Interference	−0.92	1.78	4.25	6.70
Clinic 1	1.88	–	–		0.48	2.12	2.69	5.29
Clinic 2	2.31	–	–		−0.32	2.06	3.84	5.50
Clinic 3	2.09	–	–		−0.38	2.09	4.25	5.94
Clinic 4	1.16	–	–		−1.39	1.20	2.34	4.58
Clinic 5	2.38	–	–		−0.47	2.12	3.32	5.92
Clinic 6	1.54	–	–		−0.54	1.95	3.85	4.95
Clinic 7	1.31	–	–		−0.50	2.03	4.10	5.17
Clinic 8	2.02	–	–		−0.47	0.57	3.95	5.03
Clinic 9	1.94	–	–		−0.24	2.05	3.31	5.04
Clinic 10	1.14	–	–		−0.21	1.60	3.30	4.48
Clinic 11	2.23	–	–		0.10	1.96	4.83	5.79
Clinic 12	1.92	–	–		−0.06	1.62	4.13	5.19
Clinic 13	2.44	–	–		0.05	1.65	3.72	4.83
Clinic 14	2.14	–	–		−0.12	2.04	4.06	5.66
Clinic 15	1.11	–	–		0.26	2.16	2.94	4.20
Clinic 16	0.53	–	–		0.75	2.61	3.70	4.82
Clinic 17	1.27	–	–		0.28	1.81	4.03	5.62
Clinic 18	1.62	–	–		0.76	2.72	3.92	5.49
Clinic 19	1.93	–	–		0.18	3.24	5.16	6.08
Clinic 20	1.99	–	–		−0.20	2.64	4.52	5.53
Clinic 21	2.88	–	–		−1.20	1.42	2.33	4.83
Clinic 22	2.52	–	–		0.51	2.78	3.90	4.98
Clinic 23	1.95	–	–		0.50	2.35	4.76	5.74
Clinic 24	0.66	–	–		−0.86	0.66	2.98	4.23
Clinic 25	1.12	–	–		0.61	1.94	3.50	5.11
Clinic 26	1.24	–	–		−0.40	1.80	3.34	5.62

**Fig. 2:**
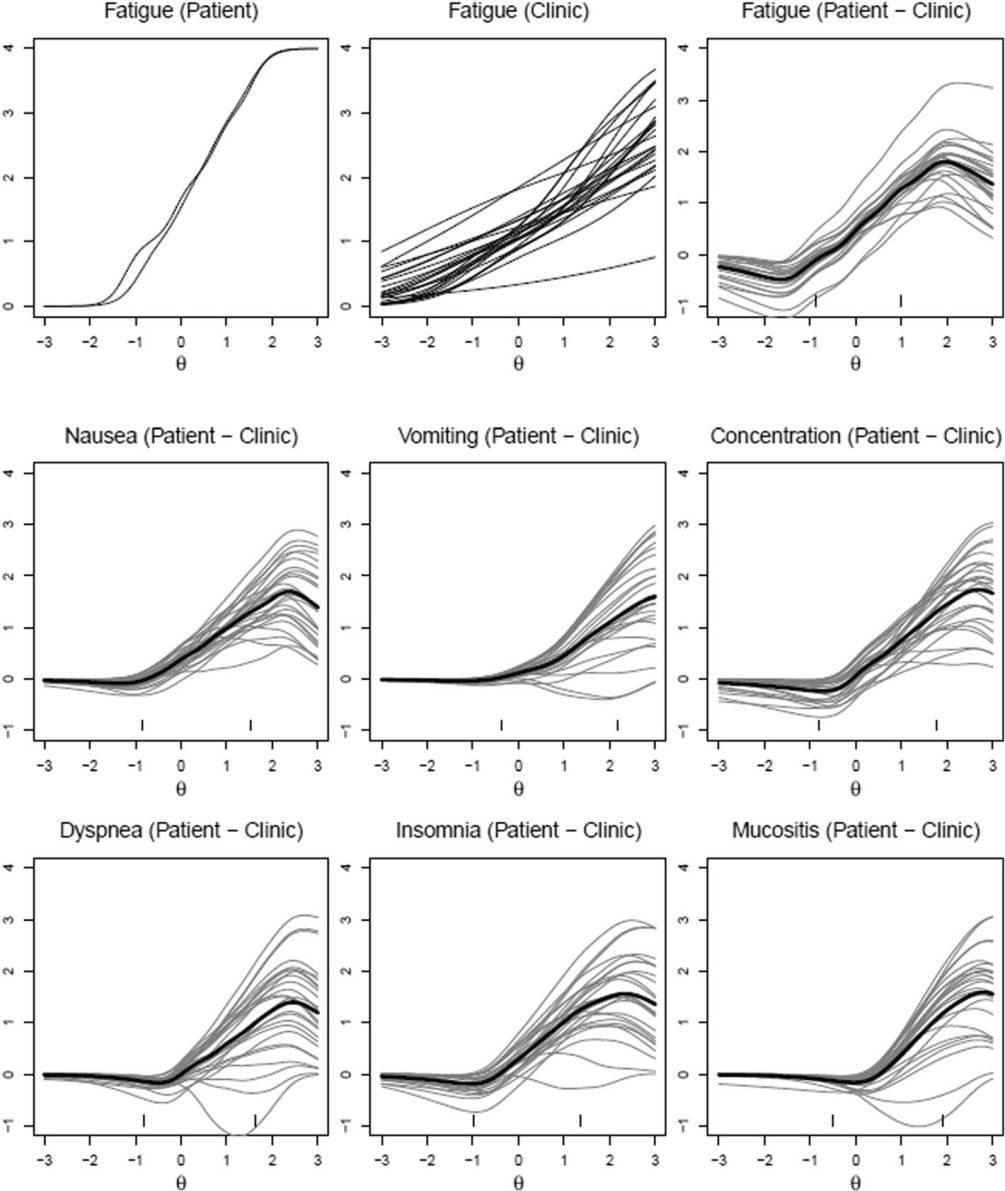
Graded Response Model Estimates for Patients/Clinicians, and Difference between Patient and Clinicians for Two-Attribute Symptoms. *Note:* X-axis represents underlying distribution of AE in the population (θ parameter in the GRM); Y-axis represents the model estimated AE ratings. In the case of fatigue, θ represents severity and interference with daily activities

Figure 2 represents the model-predicted item response curves for patients, clinics, and the resulting difference between patient and clinic ratings for the two-attribute symptoms. The upper leftmost subplot of Fig. 2 displays the severity and interference ratings for all patients for fatigue, in the same fashion as Baldwin [6] (similar to right subplot in Fig. 1). The upper center subplot of Fig. 2 illustrates the model-estimated clinic CTCAE ratings for fatigue. Each of the 45 clinics have one unique model-based expected rating profile across θ since each clinic was treated as an ‘item’ in the model. Clinics were generally sensitive to different levels of fatigue, as evidenced in the overall pattern of higher model-based ratings as θ increased. However, there was considerable variability across clinics, which would be better illustrated by taking the difference between clinic estimates and patient estimates.

The upper rightmost subplot of Fig. 2 displays the difference between patients and clinics. Details on the calculation of the differences are provided here to aid in the interpretation of the results. We extended the Bayesian GRM such that patient estimates of severity and interference with daily activities were averaged across all patients, whereas clinic estimates were calculated separately for each clinic. In the upper rightmost subplot of Fig. 2, a difference of zero represents perfect concordance, with positive and negative values representing clinic underestimation and overestimation of patient-reported AEs, respectively. The average difference between patient and clinic estimates was calculated and plotted as the thick line. The thick line shows that, generally, there is a reasonable concordance when the thick line is 1 unit above or below the value of 0 on the y-axis. For fatigue, this appears to occur between θ values of 1 or less. Clinic and patients’ expected ratings begin to diverge at theta values above 1. The divergence reaches a peak of a nearly 2-point difference when the latent fatigue is near θ = 2, 2 standard deviations above the norm.

Additionally, two tick marks are placed along the x-axis to represent two hypothetical patients; on the left, a patient (θ = − 0.9) who reported no fatigue (i.e., frequency = never), and on the right, a patient (θ = 1.0) who reported frequency as quite a bit and severity as severe. The left tick mark shows good patient-clinic concordance when fatigue is absent and the right tick mark shows clinic underestimation by approximately 1 point (on the y-axis) when fatigue is rated as occurring “quite a bit” and having “severe” severity and “very much” interference with daily activities. This difference subplot was then repeated for all two-attribute AEs and displayed within Fig. 2.

**Fig. 3:**
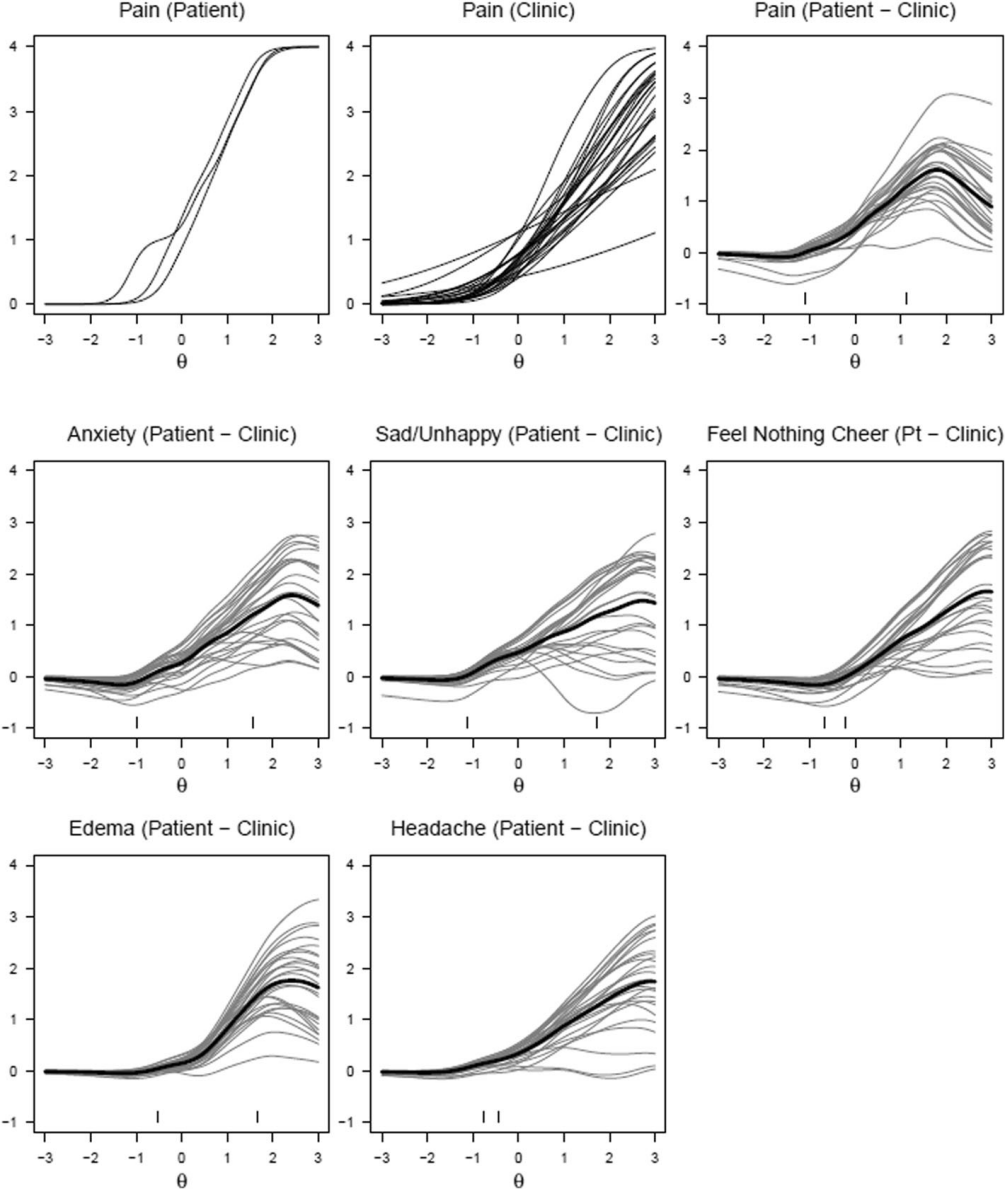
Graded Response Model Estimates for Patients/Clinicians, and Difference between Patient and Clinicians for Three-Attribute Symptoms. *Note:* X-axis represents underlying distribution of AE in the population (θ parameter in the GRM); Y-axis represents the model estimated AE ratings. In the case of pain, θ represents frequency, severity, and interference with daily activities

Figure 3 follows a similar format to Fig. 2, with GRM estimates displayed for patients, clinics, and the resulting difference between patient and clinic ratings for the three-attribute symptoms. In this case, the upper leftmost subplot of Fig. 3 displays the model-estimated frequency, severity, and interference PRO-CTCAE ratings for all patients for pain. Similar to fatigue in Fig. 2, there was an observed overlap of the multiple attribute ratings as the latent θ -values increased, suggesting that the patients’ ratings of increasing levels of pain frequency had relatively similar patterns of increased pain severity and interference of pain with daily activities.

The upper rightmost subplot represents the difference between model-estimates when subtracting clinic estimates from patient estimates, with this subplot repeated for the remaining three-attribute symptoms. In terms of pain, there is a reasonable concordance up to 1 standard deviation above the norm, as shown in the horizontal thick line near the value of zero. This good concordance only begins to slowly diverge when latent pain exceeds 1 standard deviation above the norm. The majority of clinic estimates thus agree reasonably well with patient estimates. The left tick mark shows that almost all clinic ratings agree with patient ratings when pain is absent. However, even for a patient who reports severe pain (right tick mark), the discordance remains relatively minor, generally not exceeding a difference of one grade.

Similar good concordance is seen in assessments of edema and headache. By contrast, concordance is lower for assessments of anxiety, sad and unhappy feelings, and the feeling that nothing could cheer you up.

## Discussion

The present study demonstrates a step-by-step approach to successfully applying Bayesian IRT modeling as a graphical characterization of the concordance between unidimensional clinician and multiple attribute patient ratings of symptomatic AEs. Across all AEs, it was observed that CTCAE and PRO-CTCAE ratings were more concordant at the lower model-estimated thresholds (i.e., latent AE value > 1 standard deviation below the norm), whereas there was increased discordance between clinician and patient reports as the model-estimated grading thresholds increased. This demonstrates the illustrative advantages of the IRT approach as a supplement to conventional concordance metrics, in that the IRT approach can reconcile multiple attribute ratings and better elucidate nuanced differences between independent rating sources. Specifically, Figs. 2 and 3 show how concordance between patients and clinics varies as a function of the AE level the patient is experiencing.

Conventional concordance metrics such as Cohen’s weighted ĸ or Spearman’s *r* provide a single coefficient that characterizes overall concordance between two rating sources. In the present dataset, these traditional concordance metrics were low to moderate (Table 5). If this was the only information presented, one might conclude that patient ratings deviate considerably from clinic ratings. Additionally, alternative IRT approaches such as the many-facet model [7] would assume that a single theta drives all responses; the Bayesian GRM treats them as separate pieces of information. The Bayesian GRM is a potential tool that would allow investigators to descriptively explore where discordance may arise. For instance, in the present dataset where there is low incidence of clinic assignment of elevated AE ratings.

Other methodological advantages of the Bayesian IRT approach include a pragmatic method to address missing observations. In the current PRO-CTCAE design, patients are not prompted to report symptom severity and/or interference if they first report no symptom frequency. Despite the skip pattern, the Bayesian approach can still estimate a patient-clinician difference, conditional on a latent model-based symptom distribution.

Envisioned applications of the Bayesian IRT approach include studies that compare clinician and patient ratings of PROs more broadly, beyond AEs, including those related to general health or specific symptoms. Additionally, these techniques could also be useful when making comparisons between independent ratings of HRQOL concepts. An extension of the original framework proposed by Baldwin [6], where clinical judgments were compared amongst independent clinicians, might be applied in contexts such as the assessment of HRQOL in children and adolescents, where proxy ratings are also obtained by independent teachers, clinicians, parents, or other observers via standardized checklists [31]. Additionally, this Bayesian IRT technique could be used to analyze archival datasets, such as the one described in Preen et al. [32], where multiple data sources were compared to determine the accuracy of comorbidity information.

There are a number of potential caveats that should be considered as analysts apply these methods to other study contexts where measuring extent of agreement is salient. While the Bayesian GRM was helpful in elaborating the underlying patterns of discordance between clinician and patient ratings of AEs, the sources of these differences are not thoroughly explained through this method. Additionally, in this context IRT operates under the assumption that patient and clinic ratings are locally independent given the model. A formal statistical investigation of whether such independent ratings are codependent was not made, as it is beyond the scope of this present study.

Along similar lines, a typical IRT analysis would include an evaluation of how well the model fits (e.g., infit analysis). Unfortunately because of the way the present data is structured (e.g., patients rate themselves), there is insufficient information to complete such an analysis. Future studies that plan to incorporate this Bayesian IRT technique should plan to collect necessary information to evaluate model fit. In addition, there should be caution with interpretation of the tracelines, as there may be more error around the tracelines in the right side of the symptom continuum in Figs. 2 and 3 due to sparseness of data (i.e., where respondents are reporting more severe AEs). However, the patterns observed in the IRT models are consistent with prior literature that clinicians underreport the severity of symptoms compared to patients [12, 13].

Lastly, clinician-level data was not captured by all sites during the original study, making it unclear as to which clinicians assigned ratings to which patients. As such, to apply the Bayesian GRM framework, a clinic-based aggregate variable consisting of cross-tabulations between study site and patient disease type was calculated to approximate clinic ratings of symptoms. Since it is possible that information about clinician rating variability was lost during this calculation, future studies may benefit from explicit coding of the multi-level data structure which clinicians rated each particular patient or group of patients. Given these limitations, it should be noted that the purpose of the present study was not to evaluate the linkage between CTCAE and PRO-CTCAE ratings and thus should not be considered as a commentary on the psychometric properties of PRO-CTCAE, which have been well-established elsewhere [17]. Future work that attempts to equate PRO-CTCAE ratings made by patients with CTCAE ratings assigned by clinicians should consider including cognitive interview techniques [33] to qualitatively assess patient perception of life threatening AEs and/or disease.

## Conclusions

We demonstrated that this IRT-based Bayesian GRM can be a useful descriptive tool for understanding and visualizing the features of a dataset that contained multiple attribute ratings from multiple raters (i.e., up to three ratings from a patient and a unidimensional clinician rating). This methodology can help to provide additional insight beyond that derived from traditional concordance metrics, and should be considered when the dataset of interest is amenable to such modeling techniques. Future studies examining concordance among multiple raters in the assessment of patient-, clinician-, and/or proxy-reported outcomes may benefit from the a priori incorporation of IRT into the analyses to supplement traditional concordance ratings.

## Abbreviations

AE(s): Adverse event(s)
CTCAE: Common Terminology Criteria for Adverse Events
GRM: Graded Response Model
HRQOL: Health-Related Quality of Life
IRT: Item Response Theory
NCI: National Cancer Institute
PRO(s): Patient-reported outcome(s)
PRO-CTCAE: Patient-Reported Outcomes version of the Common Terminology Criteria for Adverse Events

## Footnotes

1 For the most updated version of the PRO-CTCAE items and permission to use PRO-CTCAE, investigators should contact the Outcomes Research Branch at the National Cancer Institute (http://outcomes.cancer.gov/tools/pro-ctcae.html).

2 R/JAGS syntax for this analysis is available from the authors upon request.
